# Statistical Optimization of Process Variables for Antibiotic Activity of *Xenorhabdus bovienii*

**DOI:** 10.1371/journal.pone.0038421

**Published:** 2012-06-06

**Authors:** Xiang-Ling Fang, Li-Rong Han, Xue-Qiang Cao, Ming-Xuan Zhu, Xing Zhang, Yong-Hong Wang

**Affiliations:** Research and Development Center of Biorational Pesticides, Northwest A & F University, Yangling, Shaanxi, People’s Republic of China; University of Technology Sydney, Australia

## Abstract

The production of secondary metabolites with antibiotic properties is a common characteristic to entomopathogenic bacteria *Xenorhabdus* spp. These metabolites not only have diverse chemical structures but also have a wide range of bioactivities of medicinal and agricultural interests. Culture variables are critical to the production of secondary metabolites of microorganisms. Manipulating culture process variables can promote secondary metabolite biosynthesis and thus facilitate the discovery of novel natural products. This work was conducted to evaluate the effects of five process variables (initial pH, medium volume, rotary speed, temperature, and inoculation volume) on the antibiotic production of *Xenorhabdus bovienii* YL002 using response surface methodology. A 2^5–1^ factorial central composite design was chosen to determine the combined effects of the five variables, and to design a minimum number of experiments. The experimental and predicted antibiotic activity of *X. bovienii* YL002 was in close agreement. Statistical analysis of the results showed that initial pH, medium volume, rotary speed and temperature had a significant effect (*P*<0.05) on the antibiotic production of *X. bovienii* YL002 at their individual level; medium volume and rotary speed showed a significant effect at a combined level and was most significant at an individual level. The maximum antibiotic activity (287.5 U/mL) was achieved at the initial pH of 8.24, medium volume of 54 mL in 250 mL flask, rotary speed of 208 rpm, temperature of 32.0°C and inoculation volume of 13.8%. After optimization, the antibiotic activity was improved by 23.02% as compared with that of unoptimized conditions.

## Introduction

*Xenorhabdus* is a unique genus of bacteria symbiotically associated with entomopathogenic nematode belonging to genus *Steinernema*. The primary (1°) phase of the bacteria is carried in the intestine of the infective juvenile (IJ) developmental stage of the nematode. The IJ penetrates the insect host and releases the bacteria into the hemocoel. The bacteria multiply rapidly and produce various metabolites which can overcome the insect immune system, kill the insect, and inhibit the growth of various fungal and bacterial competitors [1]–[3]. By doing so, the bacterial symbionts are believed to prevent putrefaction of the insect cadaver and establish conditions that favor the development of both the nematode and bacterial symbionts. The antimicrobial nature of metabolites produced by *Xenorhabdus* spp. is known, and several compounds with antibiotic activity have been isolated and identified. These include indoles [4], xenorhabdins [5], xenocoumacin [6], nematophin [7], benzylineacetone [8], xenortides and xenematide [9] and cyclolipopeptide [10]. These metabolites not only have diverse chemical structures, but also have a wide range of bioactivities of medicinal and agricultural interests such as antibiotic, antimycotic, insecticidal, nematicidal, antiulcer, antineoplastic and antiviral.

*X. bovienii* has been known to produce two classes of antibiotics, indoles and dithiolopyrrolones, which could inhibit *Botrytis cinerea*, *Phytophthora capsici*, and *Phytophthora ultimum* [11]. *X. bovienii* strain A2 appears to be unusual in the diversity of small-molecules, antimicrobial compounds since xenomins and xenorxides, several xenorhabdins, including three novel ones, and four indoles have been only isolated from this strain [12]. These compounds showed strong activity against Gram-positive bacteria, yeast and many fungal species. Of particular interest is the activity shown by nematophin and xenorxides with in vitro tests against multidrug-resistant strains of *Staphylococcus aureus*. Xenorhabdins are dithiolopyrrolone derivatives, and have significant antibacterial activity against Gram-positive bacteria but have little effect against Gram-negative bacteria [5]. The xenomins and xenorxides share a similar heterocyclic ring structure with the dithiolopyrrolones except that one of the two sulphate atoms is oxidized. All of these compounds exhibit somewhat similar antimicrobial activity, but they show substantial differences in their activities in mammals, and the precise antimicrobial mode of action of these oxidized compounds has not been described. Metabolites of *X. bovienii* may offer a good opportunity for the control of diseases on plants caused by different pathogens as previous reports that metabolites of *X. bovienii* can inhibit *P. infestans* on potato [13], metabolites of *X. bovienii* SN showed 100% inhibition of *P. cactorum* lesions in pecan [14], and *X. bovienii* YL002 showed potent inhibition effect on *P. capsici* and *B. cinerea* both *in vitro* and *in vivo* [15].

Culture conditions are critical to the secondary metabolites production of microorganisms [16]. Manipulating culture variables can promote biosynthesis of the secondary metabolite and thus facilitate the discovery of novel natural products. Temperature and aeration can influence bacterial growth and, consequently, affect antibiotic production during culture [1], [3]. A much lower antibiotic activity was observed for *X. nematophilus* D1 strain cultured at 35°C than that at 15–30°C [3]. *X. nematophila* BC1 produced two-five times more nematophin over the whole period than those of strains D1 and ATCC 19061 [7]. Recent whole-genome sequencing programs have revealed that the biosynthetic potential of microorganisms has been greatly underexplored, relying as it does on traditional approaches. In fact, the number of genes encoding biosynthetic enzymes in various bacteria and fungi clearly outnumbers the known secondary metabolites of these organisms. *P. luminescens* TT01, the best studied of these bacterial symbionts, has a sequenced genome containing a wide array of antibiotic synthesizing genes with at least 33 genes in 20 loci similar to those used to make nonribosomal peptides and polyketides, two large families of biologically active small molecules [17]. The genome of *X. nematophila* ATCC 19061 has recently been sequenced, and it appears to have a similar size and comparable number of small-molecule biosynthetic genes [18]. The majority of these encoded molecules are cryptic. One reason for this observation might be that only a subset of biosynthetic pathway genes is expressed under standard laboratory culture conditions and therefore only a minority of potential chemical structures is produced. Most cryptic metabolite biosyntheses are tightly regulated, and are only activated under specific conditions. Methods to trigger biosynthetic pathways to yield cryptic natural products involve culture conditions, external cues, co-culture and genomic approaches such as genome-mining, epigenetic remodeling, and engineered pathway activation [19]. For example, by altering easily accessible culture variables such as aeration, temperature or volume, some unknown natural products were isolated from various fungi and actinomycetes [16]. There is usually a relationship between culture conditions and biosynthesis of antibiotics [3], [7], [20]–[22]. Therefore, to promote secondary metabolite biosynthesis and thus facilitate the discovery of novel natural products, it is a prerequisite to design proper culture conditions in an efficient fermentation process.

The application of statistical experimental design techniques in culture process can result in improved product yields, reduced process variability, reduced time and overall costs, compared with conventional practice of single factor optimization. Response surface methodology can be used to evaluate the relative significance of several contributing factors even in the presence of complex interactions [23], [24]. Several culture variables (initial pH, medium volume, rotary speed, temperature, and inoculation volume) have been known to affect the growth and antibiotic production of *Xenorhabdus* spp. Some studies have investigated the effects of these factors on the antibiotic activity of *Xenorhabdus* spp. by one-factor-at-a-time approach [1], [3], [20], [21], but little information exists on their combined or interactive effects. Therefore, in this study, response surface methodology (RSM) was used to optimize the culture conditions for the antibiotic production of *X. bovienii* YL002, and to evaluate the effects of variables during culture.

## Materials and Methods

### Symbiotic Bacterium Strain and Culture Conditions

No specific permits were required for the described field studies. The location is not privately-owned or protected in any way. The field studies did not involve endangered or protected species. *Xenorhabdus bovienii* YL002 was isolated from its nematode symbiont *Steinernema* sp. YL002 obtained from the soil from Yangling, China. This strain was indentified to be *X. bovienii* according to its morphological and biochemical characteristics [25], [26].

*X. bovienii* occurs in two phases (primary phase 1° and secondary phase 2°), and only primary phase 1° exhibits antibiotic activity [27], and thus primary phase 1° of *X. bovienii* YL002 was used in this study. Glycerinated stocks of this stain are deposited at the Agricultural Culture Collection Institute of the Research and Development Center of Biorational Pesticides, Northwest A & F University, China. Glycerinated stocks of this stain stored at −70°C were used as starting material for culture. To ensure the presence of primary phase 1°, the glycerinated stocks were incubated in the dark at 28°C on NA supplemented with 0.04% triphenyltetrazolium chloride (w/v) and 0.025% bromothymol blue (w/v), and fresh liquid culture were started from single blue (primary phase 1°) colonies as described [27].

Seed culture was prepared by inoculating a loopful of primary phase 1° *X. bovienii* YL002 growing on NA plate into a 250 mL flask containing 50 mL fresh YSG medium (glycerol and yeast extract at 5 and 15 g/L; 1 M-MgSO_4_, 5 mL; (NH_4_)_2_SO_4_, 2 g/L; 1 M-KH_2_OP_4_, 5 mL; 1 M-K_2_HOP_4_, 5 mL; and 1 M-Na_2_SO_4_, 10 mL), which was used in all seeding experiments. Media were adjusted to a final pH of 7.0 with 1 M NaOH solution to provide optimal condition of growth for *X. bovienii* YL002. The flasks were incubated at 28°C in darkness on an Eberbach rotary shaker at 150 rpm for 18–24 h until the optical density (600 nm) and pH readings were approximately 2.0 and 7.0, respectively. Then the seed culture was transferred into different volume sterile medium in 250 mL flask and allowed to grow for 72 h, each flask containing 8.0% inoculum of the medium volume. The cultures were centrifuged (RCF 22400 g, 20 min at 4°C) to separate the bacterial cells, and the supernatants were stored at 4°C until required.

### Assay of Antibiotic Activity

Antibiotic activity was measured by an agar diffusion plate assay with *Bacillus subtilis* [28]. Briefly, 1 mL of medium containing 10^7^–10^8^ colonies of *B. subtilis* was applied to an NA plate (90 mm). After 2 h incubation at room temperature, the supernatants of the culture were filtered sterilized (0.22 µm syringe microfilter). The filtrates (100 µL) were placed on 6-mm disk filters (Whatman 3 MM paper) and air-dried. The dried disks were put on the NA plate and incubated at 28°C for 24 h to determine the relationship between the size of the zones of inhibition of bacterial growth and the concentration of the antibiotic. Antibiotic activity was expressed as units of activity per milliliter of the supernatants, where 1 U was defined as a 1.0-mm annular clearing around the antibiotic disk. For each supernatant sample, there were six replicated plates, and there were three dried disks on each plate.

Maxwell et al. [28] confirmed the assumption that the changes in the size of the zones of inhibition (expressed as units of activity per gram of insect tissue) represented changes in antibiotic concentration, the antibiotic were extracted from insect larvae killed by *X. bovienii* by homogenizing the insects in distilled water. The assumption has been used successfully to measure the antibiotic activity of *X. nematophila* [22], [29]. So, the size of the zones of inhibition served as a measure of antibiotic titer of *X. bovienii* YL002.

### Experimental Design and Optimization by RSM

The initial pH (*X*_1_), medium volume (*X*_2_, mL), rotary speed (*X*_3_, rpm), temperature (*X*_4_, °C) and inoculation volume (*X*_5_, %) were selected to find the optimal conditions for antibiotic production of *X. bovienii* YL using CCD and RSM. The ranges and levels of the variables investigated in this study are given in Table 1. The central values (zero level) chosen for experimental design were: pH 7.0, medium volume 75 mL, rotary speed 150 rpm, temperature 28.5°C and inoculation volume 8.0%. To developing the regression equation, the test variables were coded according to the following equation:

**Table 1:** Experimental ranges and levels of the independent variables.

Variable	Parameter	Range and level				
		−2	−1	0	1	2
*X*_1_	pH	4.0	5.5	7.0	8.5	10.0
*X*_2_	Medium volume (mL)	25	50	75	100	125
*X*_3_	Rotary speed (rpm)	80	115	150	185	220
*X*_4_	Temperature (°C)	20.00	24.25	28.50	32.75	37.0
*X*_5_	Inoculation volume (%)	1.0	4.5	8.0	11.5	15.0

*x_i_* = coded value of the variable *X_i_.*

*x*_1_ = (pH–7)/1.5; *x*_2_ = (Medium volume–75)/25; *x*_3_ = (Rotary speed–150)/35;

*x*_4_ = (Temperature–28.5)/4.25; *x*_5_ = (Inoculation volume–8)/3.5.



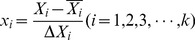
(1)Where *x*_i_ is the coded value of independent variable, *X*_i_ is the real value of independent variable, 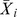 is the real value of independent variable at centre point, and Δ*X*_i_ is the value of step change. The response variable (antibiotic activity units) was fitted by a second order model in order to correlate the response variable to the independent variables. The general form of the second degree polynomial equation is:



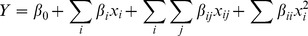
(2)Where *Y* is the measured response, *β_0_* is the intercept term, *β_i_*, *β_ij_*_,_ and *β_ii_* is the measure of the effect of variables *x_i_*, *x_i_x_j_* and *x* ^2^ *_i_*, respectively. The variable *x_i_x_j_* represents the first-order interaction between *x_i_* and *x_j_* (*i*<*j*).

For the conditions of fermentation optimization, central composite design (CCD) for five independent variables each at five levels with ten axial points and ten replicates at the center points were employed to fit the second-order polynomial model. The CCD primarily consists of a 2*^n^* ^−1^ factorial experimental design, and the total number of this experimental design is 36 (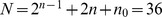)_,_ where *n* is the number of test variables, *n*_0_ is the number of center points (*n*_0_≥1), and two axial points on the axis of each design variable at a distance of 2 (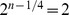, *n* = 5) from the design center. Therefore, a total of 36 experiments were required for this procedure.

The ‘STATISTICA 8.0’ software (StatSoft Inc., Tulsa, USA) was used for regression and graphical analyses of the data obtained. The response variable was assigned at low and high of the observed values for a desirability of 0 and 1, respectively, to get the overall desirability. The desirability function to get the optimal combinations of independent variables was fitted by the least square method and the 3D response graph and profile for predicted values and desirability level for factors were plotted using the same software.

The statistical analysis of the model was performed in the form of analysis of variance (ANOVA). This analysis included the Fisher’s *F*-test (overall model significance), its associated probability *P*(*F*), correlation coefficient *R*, and determination coefficient *R* ^2^ that measures the goodness of fit of regression model. The analysis also include the Student’s *t*-value for the estimated coefficients and associated probabilities *P*(t). For each variable, the quadratic models were represented as contour plots.

### Metabolite Analysis by HPLC

*X. bovienii* YL002 was cultured as above under optimized and unoptimized conditions. The cultures were vigorously extracted with 6 mL of ethyl acetate, centrifuged, and 4 mL of the top organic layer was dried for analysis. The dried ethyl acetate extracts were resuspended in 0.5 mL methanol. 50 µL of this mixture was injected for HPLC analysis in order to quantify metabolite production. Separation was performed over a Discovery RP-amide C16 (25 cm×4.6 mm, 5 µM, Supelco) HPLC column with acetonitrile by keeping water gradient at 1 mL/min: 0–2 min at 10% acetonitrile, 2–20 min at 10–40% acetonitrile, 20–40 min at 40–100% acetonitrile, 40–45 min at 100–10% acetonitrile, and 45–55 min at 10% acetonitrile.

## Results

### ANOVA and Model Fitting

In order to evaluate the relationship between response and independent variables and to determine the maximum antibiotic activity production corresponding to the optimal levels of initial pH (*X*_1_), medium volume (*X*_2_, mL), rotary speed (*X*_3_, rpm), temperature (*X*_4_, °C) and inoculation volume (*X*_5_, %), a second-order polynomial model was proposed to calculate the optimal levels of these variables. By applying multiple regression analysis on experimental data, a second-order polynomial model in coded unit explains the role of each variable and their second-order interactions on antibiotic activity. Estimated regression coefficients of the second-order polynomial model were shown in Table 2. The quadratic model of response equation in terms of coded variables is:


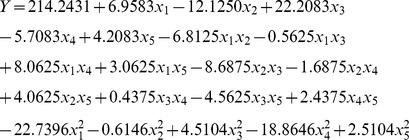
(3)

**Table 2:** Parameter estimates for factorial design experiments.

Effect	ParameterEstimate	Standarddeviation (±)	Computed*t*-value	*P*-value
Intercept	214.2431	3.525866	60.7632	0.000000
*X*_1_	6.9583	2.308221	3.0146	0.008710
*X*_1_^2^	−22.7396	1.998978	−11.3756	0.000000
*X*_2_	−12.1250	2.308221	−5.2530	0.000097
*X*_2_^2^	−0.6146	1.998978	−0.3074	0.762729
*X*_3_	22.2083	2.308221	9.6214	0.000000
*X*_3_^2^	4.5104	1.998978	2.2564	0.039403
*X*_4_	−5.7083	2.308221	−2.4730	0.025843
*X*_4_^2^	−18.8646	1.998978	−9.4371	0.000000
*X*_5_	4.2083	2.308221	1.8232	0.088264
*X*_5_^2^	2.5104	1.998978	1.2559	0.228380
*X*_1_ *X*_2_	−6.8125	2.826982	−2.4098	0.029258
*X*_1_ *X*_3_	0.5625	2.826982	0.1990	0.844954
*X*_2_ *X*_3_	−8.6875	2.826982	−3.0731	0.007731
*X*_1_ *X*_4_	8.0625	2.826982	2.8520	0.012118
*X*_2_ *X*_4_	−1.6875	2.826982	−0.5969	0.559459
*X*_3_ *X*_4_	0.4375	2.826982	0.1548	0.879075
*X*_1_ *X*_5_	3.0625	2.826982	1.0833	0.295777
*X*_2_ *X*_5_	4.0625	2.826982	1.4370	0.171236
*X*_3_ *X*_5_	−4.5625	2.826982	−1.6139	0.127381
*X*_4_ *X*_5_	2.4375	2.826982	0.8622	0.402137

Where Y is the response (antibiotic activity units) and *x*_1_, *x*_2_, *x*_3_, *x*_4_ and *x*_5_ are the coded values of the independent variables, viz. initial pH, medium volume, rotary speed, temperature, and inoculation volume, respectively. The quadratic model in Equation (3) with 21 terms contains five linear terms, five quadratic terms and ten two factorial interactions. After removing the insignificant terms (on the basis of *P*-values which were >0.05), the model Equation (3) was reduced to the following fitted model Equation:


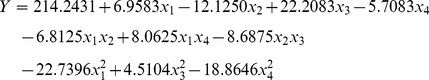
(4)

**Figure 1:**
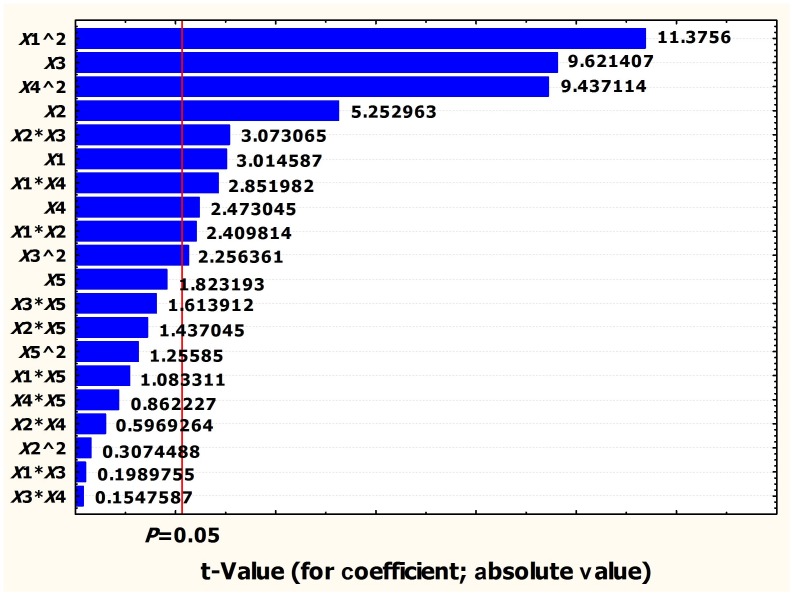
Pareto chart of *t*-values for coefficients.

Quadratic response surface regression designs are a hybrid type of design with characteristics of both polynomial regression designs and fractional factorial regression designs, which contain all the same effects of polynomial regression designs to degree 2 and additionally the 2-way interaction effects of the predictor variables. Each effect could be estimated independently due to the orthogonal design. The ANOVA result was shown in Table 3. The linear term of *X*_1_, *X*_2_, *X*_3_ and *X*_4_ were significant at *P*<0.05 level. Among the linear terms, the main effects of medium volume and rotary speed on the antibiotic activity were most significant as was evident from their respective *P*-values (*P_X_*_2_ = 0.000097 and *P_X_*_3_ = 0.0000). Temperature also had significant effect on the antibiotic activity at the linear term (*P_X_*_4_ = 025843). Partial eta squared was used as a measure of effect size. Rotary speed had highest effect on the antibiotic production as given by highest linear partial eta squared (0.860558), followed by medium volume (0.647835), pH (0.377276) and temperature (0.289636). The Pareto chart showed each of the estimated effects, interactions and the standard deviation of each of the effects which measures their sampling error (Fig. 1). In the experimental design the Pareto chart is a Frequency Histogram that shows the amount of effect of each factor has on the response in decreasing order, and often, a line going across the columns indicates how large an effect has to be (i.e., how long a column must be) to be statistically significant. Quadratic term of *X*_1_^2^, *X*_3_^2^ and *X*_4_^2^ (pH, rotary speed and temperature) were significant *P*<0.05. The result also indicated that pH, rotary speed and temperature could act as limiting factors, and small variations in their values will considerably alter either growth rate or product formation rate or both. Interactive terms of *X*_1_ *X*_2_, *X*_1_ *X*_ 4_ and *X*_2_ *X*_3_ were also significant at *P*<0.05. The interaction between medium volume and rotary speed had significant effect on antibiotic production (*P_X_*_2*X*3_<0.007731). These results suggested that medium volume and rotary speed had a direct relationship with antibiotic activity under this condition.

**Table 3:** Analysis of variance for parameter estimates for factorial design experiments.

Effect	SS	DF	MS	*F*-value	*P*-value	Partial eta-squared	Non-centrality	Observed power (alpha = 0.05)
Intercept	472115.2	1	472115.2	3692.172	0.000000	0.995954	3692.172	1.000000
*X*_1_	1162.0	1	1162.0	9.088	0.008710	0.377276	9.088	0.804329
*X*_1_^2^	16546.8	1	16546.8	129.404	0.000000	0.896125	129.404	1.000000
*X*_2_	3528.4	1	3528.4	27.594	0.000097	0.647835	27.594	0.998327
*X*_2_^2^	12.1	1	12.1	0.095	0.762729	0.006262	0.095	0.059573
*X*_3_	11837.0	1	11837.0	92.571	0.000000	0.860558	92.571	1.000000
*X*_3_^2^	651.0	1	651.0	5.091	0.039403	0.253403	5.091	0.559968
*X*_4_	782.0	1	782.0	6.116	0.025843	0.289636	6.116	0.637905
*X*_4_^2^	11387.9	1	11387.9	89.059	0.000000	0.855851	89.059	1.000000
*X*_5_	425.0	1	425.0	3.324	0.088264	0.181403	3.324	0.400112
*X*_5_^2^	201.7	1	201.7	1.577	0.228380	0.095141	1.577	0.217510
*X*_1_ *X*_2_	742.6	1	742.6	5.807	0.029258	0.279096	5.807	0.615596
*X*_1_ *X*_3_	5.1	1	5.1	0.040	0.844954	0.002632	0.040	0.053997
*X*_2_ *X*_3_	1207.6	1	1207.6	9.444	0.007731	0.386346	9.444	0.819011
*X*_1_ *X*_4_	1040.1	1	1040.1	8.134	0.012118	0.351598	8.134	0.759853
*X*_2_ *X*_4_	45.6	1	45.6	0.356	0.559459	0.023204	0.356	0.086576
*X*_3_ *X*_4_	3.1	1	3.1	0.024	0.879075	0.001594	0.024	0.052416
*X*_1_ *X*_5_	150.1	1	150.1	1.174	0.295777	0.072561	1.174	0.173808
*X*_2_ *X*_5_	264.1	1	264.1	2.065	0.171236	0.121013	2.065	0.270040
*X*_3_ *X*_5_	333.1	1	333.1	2.605	0.127381	0.147955	2.605	0.327032
*X*_4_ *X*_5_	95.1	1	95.1	0.743	0.402137	0.047222	0.743	0.127504
Error	1918.0	15	127.9					

The significance of effects was determined by Student’s *t*-test and *P*-value (Table 2). The larger the magnitude of *t*-test and smaller *P*-value, the more significant is the corresponding effect. The positive effect of *X*_1_ and *X*_3_ indicated a linear effect to increase antibiotic activity while negative effect of *X*_2_ and *X*_4_ showed a linear effect to decrease antibiotic activity. The negative effect of *X*_1_^2^ and *X*_4_^2^ indicated that the antibiotic production increased as the level of these factors increased and decreased as the level of these parameters increased above certain values.

The ANOVA of the quadratic regression model showed that the model was highly significant, as was evident from the low *P* value of the Fisher’s *F*-test (*F*_model_, mean square regression/mean square residual = 35.39577) [(*P*_model_>*F*) = 0.0000] (Table 4). This proved that the model equation as expressed in Equation (4) provides a suitable model to describe the response of the experiment pertaining to antibiotic activity. The model also showed a statistically insignificant lack of fit, as was evident from the lower calculated *F*-value (1.99) than the tabulated *F*-value (*F*_0.01(16,9)_ = 2.99) even at the 0.01 confidence level. The model was found to be adequate for prediction within the range of variables employed. The closer the *R* ^2^ value is to 1.00, the stronger the model is and the better it predicts the response [30]. In this case, the coefficient of determination *R* ^2^ = 0.93403, which implied that antibiotic activity was attributed to the given independent variables. The *R* ^2^ also indicated that only 6% of the total variations were not explained by the model. These measures indicated that the accuracy and general ability of the polynomial model was good and that analysis of the response trends using the model was reasonable. The high value of the correlation coefficient, *R* = 0.966452, indicated a good agreement between the experimental and predicted values of antibiotic activity.

**Table 4:** Analysis of variance for the quadratic model.

Source of variations	DF	SS	MS	*F*-value	*P*-value
Model	10	48885.450	4888.545	35.39577	0.0000
Residual	25	3452.774	138.1110		
Lack of fit	16	2692.374	168.2734	1.991663	0.147895
Pure error	9	760.400	84.48889		
Total	35	52338.224			

*R* ^2^ = Coefficient of determination = 0.934030; *R* = Coefficient of correlation = 0.966452.

**Figure 2:**
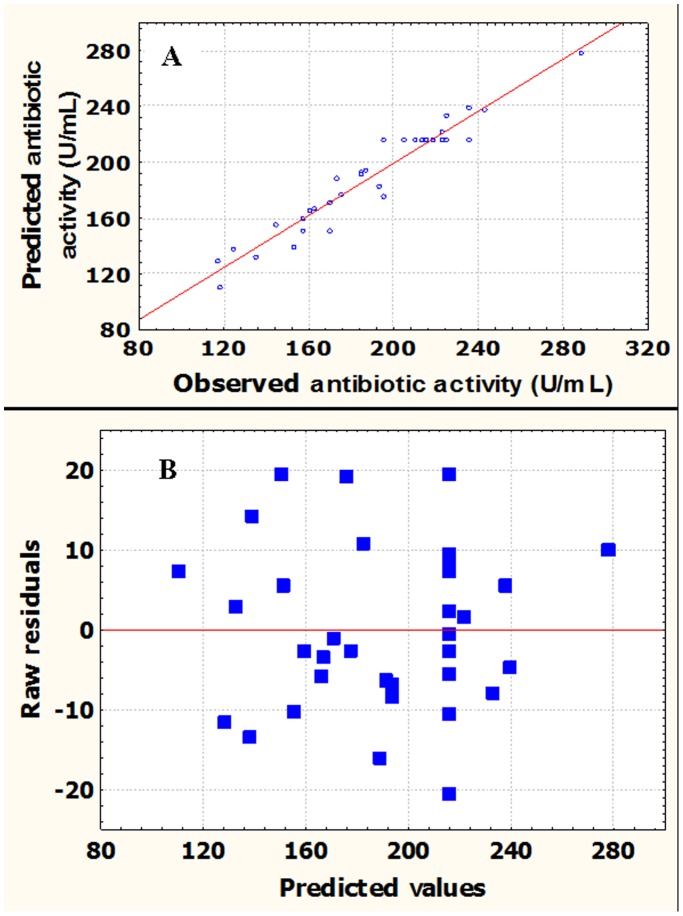
Residual diagnostics of contour surface of the quadratic model. (A) The predicted vs. observed antibiotic activity of *Xenorhabdus bovienii* YL002. (B) Plot of internally studentized residuals vs. predicted responses.

A regression model can be used to predict future observations on the response *Y* (antibiotic activity) corresponding to particular values of the variables. In predicting new observations and estimating the mean response at a given point, one must be careful about extrapolating beyond the region containing the original observations. It is very possible that a model that fits well in the region of the original data will no longer fit well outside the region [31]. Fig. 2A showed the observed antibiotic activity versus the predicted ones from the model Equation (2). Point above or below the diagonal line represented areas of over or under prediction. There were no significant violations of the model, and the predicted data of the response from the empirical model was in agreement with the observed ones in the range of the operating variables. It is necessary to check the fitted model to ensure that it provides an adequate approximation to the real system. Unless the model shows an adequate fit, proceeding with the investigation and optimization of the fitted response surface likely give poor or misleading results [31]. The residuals from the least squares fit play an important role in judging model adequacy. The assumptions for randomness, normality and constant variances of the residuals were all verified by the normal probability plot and the residual plot. A linear pattern demonstrated normality in the error term and there were no signs of any problems in our data. Fig. 2B showed a plot of residuals versus the predicted response, where residual scatters were randomly displayed, suggesting that the variance of the original observation was constant for all values of *Y*.

### Response Surface Analysis

The 3D response surface and the 2D contour plots described by the regression model were drawn to illustrate the effects of the independent variables, and interactive effects of each independent variable on the response variables.

**Figure 3:**
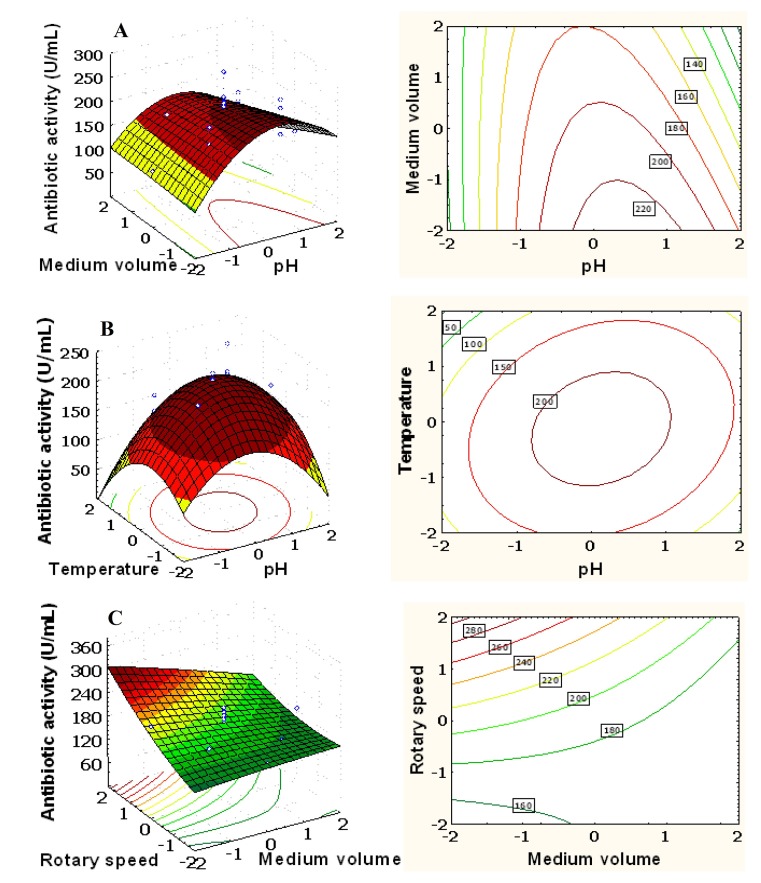
Response surface plot and contour plot. (A) The combined effect of pH and medium volume on the antibiotic activity of *Xenorhabdus bovienii* YL002. (B) The combined effect of pH and temperature on the antibiotic activity of *X. bovienii* YL002. (C) The combined effect of medium volume and rotary speed on the antibiotic activity of *X. bovienii* YL002.

Fig. 3A showed the 3D plot and its corresponding contour plot of the effects of initial pH and medium volume on the antibiotic activity while the other three variables were fixed at their middle level. With the increase of the initial pH from 4.0 to 7.0, the antibiotic activity significantly increased from 98.0 to 231.6 U/mL at a low medium volume, but only increased from 100.0 to 180.0.0 U/mL at a high medium volume. This suggests that increasing the initial pH within the tested range was beneficial to the antibiotic production. Our results also showed when the initial pH beyond 7.6, the antibiotic activity decreased. The three-dimensional plot and its respective contour plot facilitated the identification of the optimal levels of initial pH and medium volume. The optimal initial pH was around 7.2, and the medium volume was around 25.0 mL.

Fig. 3B showed the 3D plot and its corresponding contour plot of the effects of initial pH and temperature on the antibiotic activity while the other three variables were fixed at its middle level. When initial pH was near neutral, the increase of the temperature from 20.0 to 28.5°C resulted in increased antibiotic activity, and any further increase in temperature resulted in decreased antibiotic activity. However, no significant effect of temperature on antibiotic activity was observed at alkaline and acidic medium condition. The optimal temperature was around 28.5°C.

Fig. 3C showed 3D plot and its corresponding contour plot of the effects of medium volume and rotary speed on the antibiotic activity while the other three variables were fixed at its middle level. There was a significant mutual interaction between medium volume and rotary speed. At moderate initial pH and temperature, the antibiotic activity increased with the increase of rotary speed, and decreased with the increase of medium volume. At a low medium volume, the antibiotic activity steadily increased when rotary speed increased. No significant effect of rotary speed on antibiotic activity was observed at high level of medium volume.

### Optimization of Response

**Figure 4:**
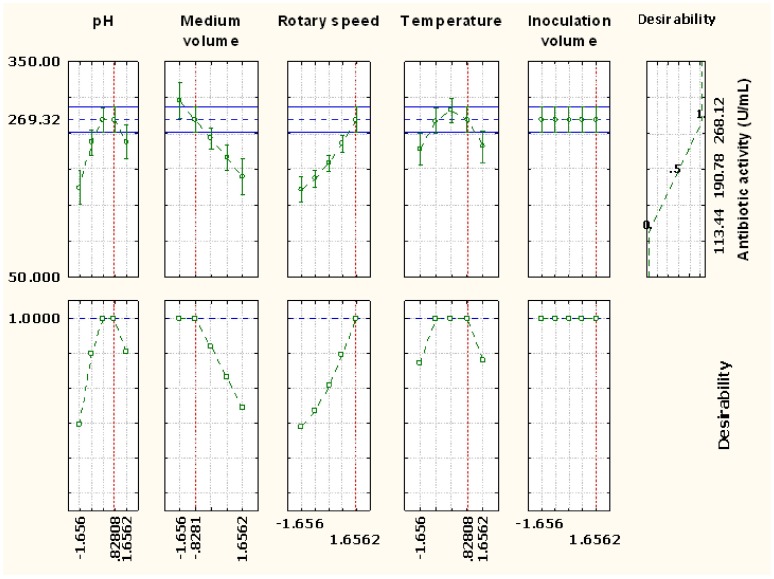
Desirability charts of variables for maximum response.

The desirability function to obtain an optimal antibiotic activity was fitted by the least square method assigning the antibiotic activity at the observed low (113.44 U/mL) and high (268.12 U/mL) values for a corresponding desirability of 0 and 1, respectively, and the profiles were plotted. These desirability profiles show which levels of predicted variables (viz. *X*_1_, *X*_2_, *X*_3_, *X*_4_ and *X*_5_) produce the most desirable predicted responses on the dependent variable (*Y*). The profiles for predicted response and the desirability level for variables (Fig. 4) indicated that pH of 8.24, medium volume of 54 mL, rotary speed of 208 rpm, temperature of 32°C and inoculation volume of 13.8% gave maximum antibiotic activity at an optimal desirability score of 1 (Fig. 4). These profiles also suggested that an increase in the pH, medium volume, rotary speed, temperatures and inoculation volume above the optimized levels will decrease the antibiotic activity.

The validation of the model was determined by carrying out experiments under the predicted optimal conditions. The experimental antibiotic activity obtained was 287.5 U/mL, which was in close agreement with the model prediction of 268.12 U/mL. Therefore, the model developed was considered to be accurate and reliable for predicting the antibiotic production of *X. bovienii* YL002. After optimization, the antibiotic activity was improved by 23.02% as compared with that obtained under unoptimized conditions (233.7 U/mL) (Table 5). Moreover, further experiments were carried out in 5 L bioreactors (Eastbio Co. Ltd., China) under the initial pH, temperature, inoculation volume and aeration speed as the rotary speed obtained from optimization study in flasks, but medium volume was kept 75% of the volume of bioreactors based on the recommended volume of the manufacturer. The antibiotic activity obtained from the bioreactor experiments was in close agreement with that of the model prediction and the flask experiments.

**Table 5:** Experimental validation of the combined effect of variables under unoptimized and optimized conditions on the antibiotic activity *Xenorhabdus bovienii* YL002.

Variable	Parameter	Level		Antibiotic activity (U/mL)		
		Unoptimized	Optimized	Unoptimized	Optimized (Predicted)	Optimized (Experimental)
*X*_1_	pH	7.0	8.24	233.7	268.1	287.5
*X*_2_	Medium volume (mL)	50.0	54.3			
*X*_3_	Rotary speed (rpm)	150	208			
*X*_4_	Temperature (°C)	28.0	32.0			
*X*_5_	Inoculation volume (%)	8.0	13.8			

**Figure 5:**
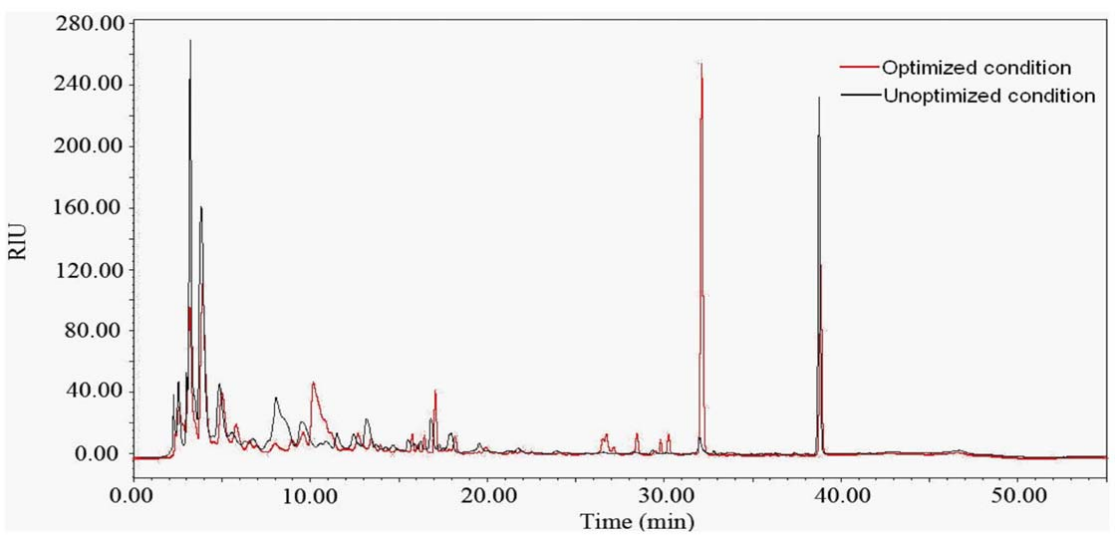
Differential metabolite profiles. *Xenorhabdus bovienii* YL002 was cultured under optimized condition and unoptimized condition, and organic extracts were assessed by high-pressure liquid chromatography (280 nm).

Metabolite profile of organic extracts from *X. bovienii* YL002 under both optimized condition and unoptimized condition by HPLC revealed that the production of metabolites was regulated by culture conditions. The metabolite profile at 32 min under optimized condition was significantly up regulated compared with that under unoptimized condition while it was slightly down regulated at 40 min (Fig. 5).

## Discussion

In this study, we focused on the optimization of the culture conditions for the antibiotic production of *X. bovienii* YL002 through factorial design using response surface analysis. The approach allowed the determination of the culture conditions that give the maximum antibiotic activity of *X. bovienii* YL002. Suitable model was found to describe the response of the experiments pertaining to the antibiotic production where the values obtained experimentally are in accordance with the expected values determined by the model. The model was validated by comparing the observed and predicted values at the optimal point. The optimization procedure allowed an increase in the antibiotic activity of *X. bovienii* YL002.

Antibiotic production of *Xenorhabdus* spp. is generally an aerobic process [1], [3]. Dissolved oxygen is an important factor in the culture process of *X. bovienii*. In shaken flasks, oxygen supply is related to medium volume and rotary speed. Decreasing medium volume or increasing rotary speed can improve the dissolved oxygen level in medium. In this study, medium volume and rotary speed were found to be the most important variable influencing antibiotic activity of *X. bovienii* YL002 at individual level and showed a significant influence at their interactive level. The maximum antibiotic activity was achieved when the rotary speed was kept at 220 rpm. Antibiotic activity increased with the decrease of culture volume or the increase of rotary speed, which indicated antibiotic production of *X. bovienii* YL002 could be improved under high DO level.

This study showed that initial pH of the culture had significant effect on antibiotic activity of *X. bovienii* YL002. It has been shown that initial pH of the culture play a crucial role in the secondary metabolite production of *Xenorhabdus* spp. [19], [20]. One possible reason is that the key enzymes that dominate antibiotic synthesis become inactivated or degenerated. Global regulators, which affect the transcription of gene ensembles via regulatory cascades, typically govern the production of small molecules in bacteria [31]. Identification and manipulation of these global regulators could provide a powerful approach to complete sets of biologically important and previously uncharacterized small molecules. A global transcriptional regulator, HexA, was associated with the secondary metabolite production of *P. luminescens*. *P. luminescens* Δ*hexA* mutant led to dramatic up regulation of biosynthesized small molecules [32]. The CpxRA signal transduction system is involved in the pathogenic and mutualistic interactions of the entomopathogenic acterium *X. nematophila* [33]. CpxA, a sensor histidine kinase with autokinase, phosphotransfer, and phospho-CpxR phosphatase activity, is located in the cytoplasmic membrane, where it senses diverse signals, including alkaline pH. In response, CpxA autophosphorylates and donates its phosphoryl group to CpxR, the cognate response regulator. When phosphorylated, this transcription factor controls part of the envelope stress response system, pilus assembly, type III secretion, motility and chemotaxis, adherence, and biofilm development [34]. CpxR negatively influences the antibiotic activities of *X. nematophila* [33]. So, CpxR-phosphate negatively regulates the antibiotic activities of *X. nematophila*. CpxP was identified as an alkaline-induced member of the Cpx regulation. This periplasmic chaperone binds to the periplasmic domain of CpxA and inhibits its autokinase activity [34]. In this study the optimal pH for antibiotic production of *X. bovienii* YL002 is 8.24, the alkaline pH may induced the production of CpxP and inhibits CpxA autophosphorylates and CpxR phosphorylates, and thus, the antibiotic activity of *X. bovienii* YL002 was enhanced.

This study also showed that temperature had significant effect on antibiotic activity of *X. bovienii* YL002. It is widely accepted that secondary metabolism of microorganisms represents an important pathway for survival. The low temperature could be viewed as one type of environmental pressures, and secondary metabolism production might therefore be enhanced [35]. In this study, the average antibiotic activity of *X. bovienii* YL002 was 214.91 U/mL when the initial pH, medium volume, rotary speed, temperature and inoculation volume were kept at its middle level. But, the antibiotic activity of *X. bovienii* YL002 was only 157.0 U/mL and 117.9 U/mL when it was cultured at 20.0°C and 37.0°C. This indicates that high and low temperatures are not beneficial to the antibiotic production of *X. bovienii* YL002. The optimal temperature may induce the associated genes expression and/or activate the key enzymes that dominate antibiotic synthesis of *X. bovienii* YL002.

It is also important to provide an optimal inoculum level in the culture of *X. bovienii* YL002. A low inoculum density may give insufficient biomass causing reduced product formation, whereas a high inoculum may produce too much biomass leading to the poor product formation [36]. However, in this study, the inoculation volume had no significant effect on antibiotic activity of *X. bovienii* YL002, which is consistent with the previous report that inoculation volume had no significant effect on the antibiotic activity of *Xenorhabdus* sp. D43 [21].

The optimization of the culture conditions of *X. bovienii* YL002 not only increased the antibiotic activity, but also significantly altered the metabolite profile. However, the relationship between the increase of antibiotic activity and up regulation of the metabolites of *Xenorhabdus* spp. and whether the optimization of culture conditions could reveal previously cryptic metabolites have not been revealed yet. In order to clarify these questions, further work will be conducted on characterization of the metabolites of *X. bovienii* YL002 under optimized condition.

### Conclusions

The optimal conditions for the culture of *X. bovienii* YL002 were at the initial pH of 8.24, medium volume of 54.3 mL in 250 mL flask, rotary speed of 208 rpm, temperature of 32.0°C, and inoculation volume of 13.8%. Maximum antibiotic activity (287.5 U/mL) was obtained under the optimized conditions compared with that of unoptimized conditions (233.7 U/mL). The chosen methods were proved to be a powerful tool for the optimization of culture conditions for antibiotic production of *X. bovienii* YL002. Furthermore, the information obtained is considered fundamental and useful for the development of *X. bovienii* YL002 culture process for efficient production of antibiotic on a large scale.
